# “Osteogenesis Imperfecta Patients Wish Orthopedic Surgeons Had Better Strategies to Help with…”—Results of a Patient and Parent-Oriented Survey

**DOI:** 10.3390/children10081345

**Published:** 2023-08-04

**Authors:** Jill Flanagan, Laura Tosi, Erika Carter, Tracy Hart, Jeanne Franzone, Maegen Wallace

**Affiliations:** 1 Department of Orthopaedic Surgery, Children’s Healthcare of Atlanta, Atlanta, GA 30329, USA; 2 Department of Orthopaedic Surgery, Children’s National, Washington, DC 20001, USA; 3 Osteogenesis Imperfecta Foundation, Gaithersburg, MD 20878, USA; 4 Department of Orthopaedic Surgery, Nemours Children’s Hospital, Wilmington, DE 19899, USA; 5 Department of Orthopaedic Surgery, Children’s Hospital and Medical Center, Omaha, NE 68114, USA; 6 Department of Orthopaedic Surgery, University of Nebraska Medical Center, Omaha, NE 68198, USA

**Keywords:** Osteogenesis Imperfecta, patient-reported outcomes, survey, orthopaedic management

## Abstract

Osteogenesis Imperfecta (OI) is a rare genetic disorder in Type I collagen characterized by bone fractures, fragility, and deformity. Current treatments are focused on decreasing fracture rates, improving bone strength, and improving overall global function. Recent research has focused primarily on fracture fixation and outcomes of intramedullary rodding of long bones. While surgical techniques continue to evolve, recent trends in OI research are focusing on patient quality of life and patient-reported outcomes. We created a 12-question survey seeking information regarding aspects of orthopedic care that OI patients and families feel are the most pressing to improve. The survey was electronically administered, and 341 individuals participated. A total of 75% of respondents who answered the age question (254/335) were adults. Regarding surgical intervention for long bones, only 16% of respondents recall being told they could not have surgery because they were too young. Of the 16%, 37.8% were told that <5 years was too young, 13.4% <4 years was too young, and 48.8% <3 years of age was too young for surgical intervention for fractures or deformities. Nearly 22% of respondents were told that their bones were too small for intramedullary fixation. The patient and family responses help elucidate the topics requiring focus for the improvement of OI orthopedic care. Patient concerns and insights should drive the research questions we ask to advance the orthopedic care of OI patients.

## 1. Introduction

Osteogenesis Imperfecta (OI) is a rare genetic disorder that occurs in 1 in 10,000 live births [1,2,3,4]. Approximately 85% of patients with OI have defective or inadequate Type I collagen characterized by bone fractures, fragility, and deformity. The other 15% of patients with OI have another genetic cause in a variety of genes that encode proteins that are involved in type I collagen synthesis or processing, or a protein that regulates the activity of bone-forming cells. As type I collagen is widespread throughout the body, many other organ systems may be involved. This may include impaired hearing, joint laxity and instability, dental issues, blue sclera, and cardiopulmonary abnormalities [5,6,7,8,9].

Current treatment methods are focused on decreasing fracture rates, improving bone strength, and improving overall global function [7,8,9]. Recent orthopedic surgery research has focused on fracture fixation and success rates after intramedullary rodding of long bones [10,11,12,13]. While surgical techniques continue to evolve and improve, recent trends in OI research are also focusing on patient quality of life and patient-reported outcomes [14,15,16,17,18,19] measures. Further, given that OI manifests early in life the experiences and roadblocks faced by parents and caregivers of children with OI present an important context for clinicians and researchers involved in the treatment of pediatric bone disorders.

In the Key4OI initiative, https://www.key4oi.org/ (accessed on1 April 2023), a collaborative investigation into quality-of-life care for patients with OI, suggest that OI patients are interested in outcomes such as self-care, pain, fatigue, upper and lower limb function, stature, fracture rates and healing, and emotional well-being [19]. In an interview with adult OI patients before the 13th International Congress on OI in Oslo, Norway, Swezey et al. [18] led discussions with leaders from the adult OI community. From their meetings, adults stressed that breathing, hearing, and age-related issues become more significant concerns rather than fractures as they get older. Similarly, the current investigators at large OI centers were interested in learning the current trends within pediatric care of OI across a wide landscape of patients with varied access to multidisciplinary care. We sought to learn more about advice regarding the timing of rodding surgery, access to water therapy, and emotional stress around fractures and surgery via a survey distributed to a large mailing list of OI patients and their families.

Moreover, we wanted to learn from the OI community we serve what their care-related priorities are, in order to improve treatment in this vulnerable patient population. Therefore, three OI surgeons collaborated with the Osteogenesis Imperfecta Foundation (OIF) to survey patients and parents that communicate with the OI Foundation and the Osteogenesis Imperfecta Federation Europe (OIFE). The survey administered seeks to identify current trends in OI care and OI access to care and help identify future research avenues and opportunities for improved clinical protocols.

## 2. Materials and Methods

**Figure 1:**
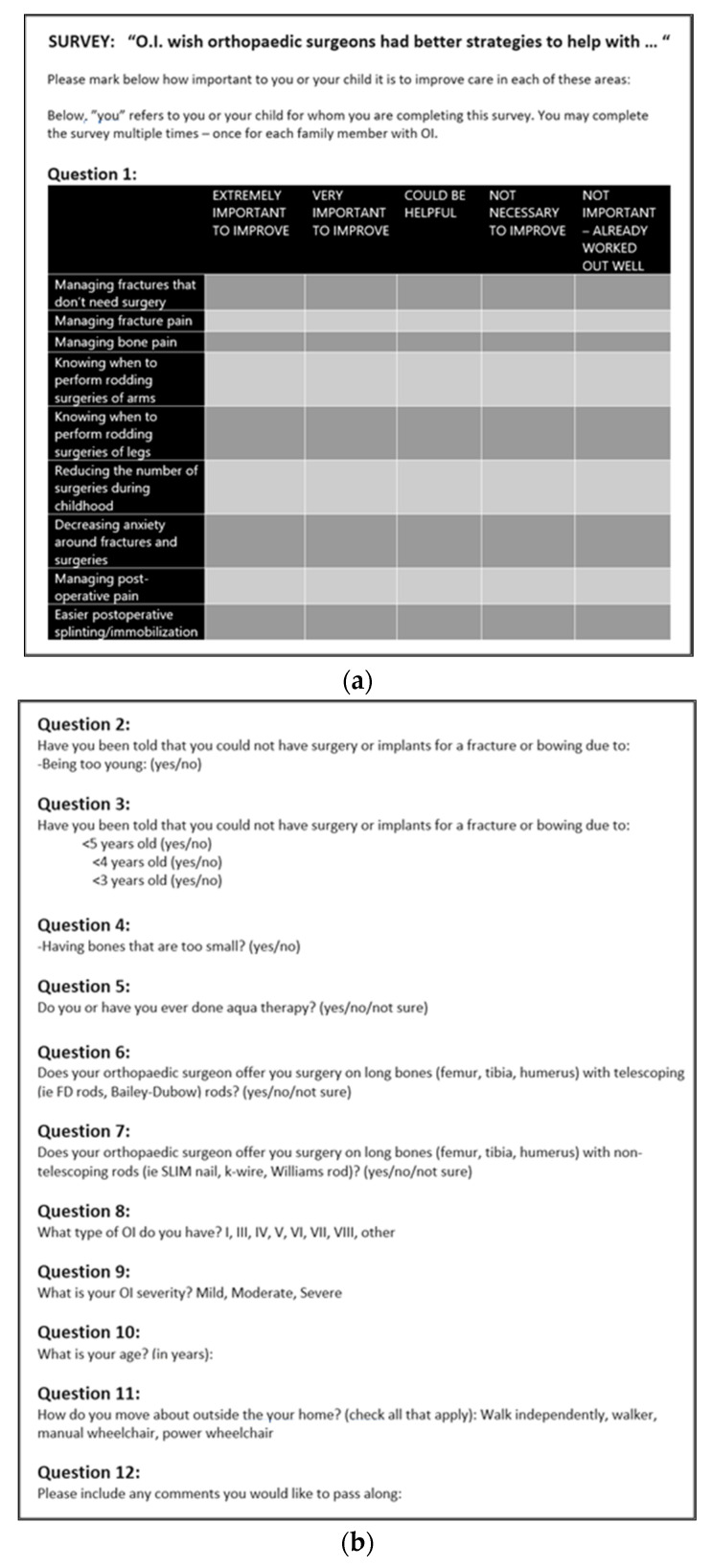
(**a**,**b**) The 12 Question survey distributed to enrollees of the OI registry.

We created a 12-question survey (Figure 1a,b) piloted by the authors and approved by the OIF. The survey was titled, “O.I. wish orthopedic surgeons had better strategies to help with…” and sought to gather information regarding the aspects of orthopedic care OI patients and families feel to be the most pressing to address. The survey was electronically distributed via Survey Monkey to all members of the OI Registry, which includes 2099 US and international enrollees. After distribution, a total of 341 individuals completed the survey. The OI Registry is housed and managed by the Health Informatics Institute at the University of South Florida, which also provided IRB approval for the survey. The first 11 questions were each analyzed into frequency of categorical answers and then graphed for ease of interpretation. As this study aims to present qualitative data from a survey study, statistical methods are limited to basic frequencies, percentages, and averages of appropriate responses. As there were no comparison groups, no additional statistical analyses were needed for this qualitative, survey-based research. All data reported in this manuscript is participant-reported, including OI type and OI severity—with the explicit aim of achieving a better understanding of *patient-perceived* OI severity. The final question of the survey indicated, “Please include any comments you would like to pass along”. To report on the responses for this question, the answers were grouped into eight different themes of answers. We report on the frequency of responses within each theme.

## 3. Results

### 3.1. Survey Participants

A total of 341 individuals completed the “O.I. wish orthopedic surgeons had better strategies to help with…” survey. A total of 336 (98.5%) individuals responded to the question regarding age in years: Average age of respondents was 31.7 years (Range 0–76 years, standard deviation 26.0 years). A total of 340 (99.7%) participants replied to the question regarding OI Type: 134 (39.4%) Type 1, 75 (22.1%) Type III, 61 (17.9%) Type IV, 9 (2.6%) Type V, and 9 (2.6%) Types VI-VIII, 12 (3.5%) Other and 40 (11.7%) Not Sure. 339 (99.4%) participants replied to the question regarding OI severity: 121 mild (35.7%), 168 moderate (49.6%), and 50 severe (14.8%). 337 (98.8%) Individuals responded to the question regarding mobility at home: 206 (61.1%) walk independently, 59 (17.5%) use a walker, 100 (29.7%) use a manual wheelchair, and 57 (16.9%) use a power wheelchair. A total of 53.4% of survey participants have participated in aquatherapy, 42.4% of respondents have not participated in aquatherapy, and 4.1% are unsure regarding participation in aquatherapy.

### 3.2. Areas of Importance to Improve Care

**Figure 2:**
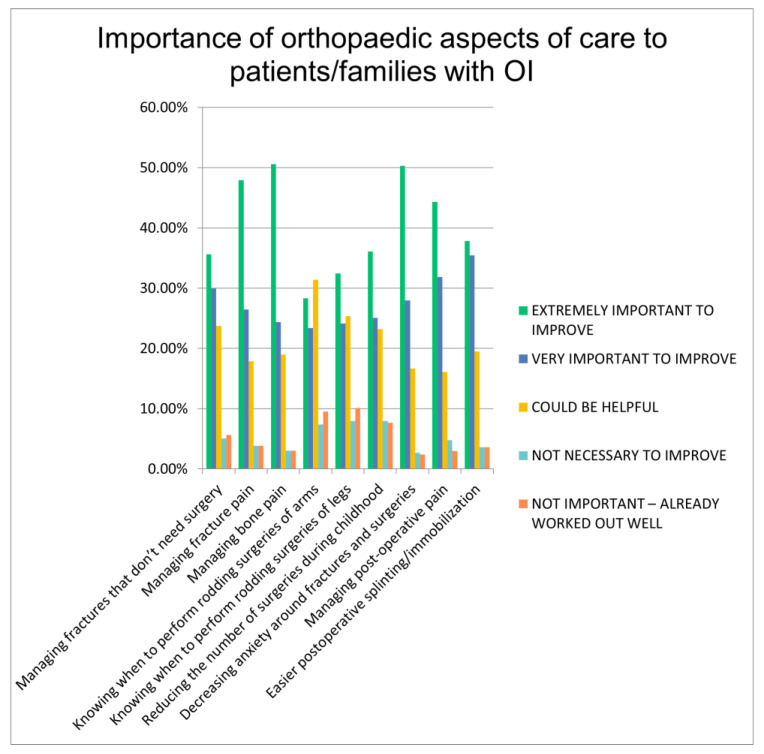
Importance of orthopedic aspects of care to patients/families with OI.

Respondents were queried about the importance of improving care in nine clinical areas: Managing fractures that do not need surgery, managing fracture pain, managing bone pain, knowing when to perform rodding surgeries of the arms, knowing when to perform rodding surgeries of the legs, reducing the number of surgeries during childhood, decreasing anxiety around fractures and surgeries, managing postoperative pain, and postoperative splinting/immobilization options. The responses are demonstrated in Figure 2. The most important aspects of orthopedic care that patients wanted surgeons to focus on revolve around pain and mental health—with managing fracture pain, bone pain, and post-op pain of the highest priorities, as well as management of anxiety surrounding fractures and surgeries.

### 3.3. Information Regarding Extremity Realignment and Rodding

A total of 16.0% of respondents have been told surgery using intramedullary implants to address a fracture or bowing of an extremity could not be performed due to being too young. Of those respondents, this occurred in the age range of less than five years old for 37.8%, less than four years old for 13.4%, and less than three years old for 48.8%. A total of 21.8% of respondents have been told surgery using intramedullary implants to address a fracture or bowing of an extremity could not be performed due to having bones that are too small.

A total of 32.6% of survey participants report their treating orthopedic surgeon offers surgery on the long bones of the extremities (femur, tibia, humerus) with telescoping intramedullary rods (examples include Fassier–Duval rods, Bailey–Dubow rods), 40.4% of survey participants report their treating orthopedic surgeon does not offer such surgery and 27.0% of respondents replied “not sure” regarding such surgery. 34.4% of respondents report their treating orthopedic surgeon has offered surgery on the long bones of the extremities (femur, tibia, and humerus) with non-telescoping rods, 31.4% of respondents report their treating orthopedic surgeon does not offer such surgery, and 34.2% of respondents replied “not sure” regarding such surgery.

### 3.4. Aquatherapy

Water therapy is an ideal medium for patients with OI to work on muscle strengthening, joint ROM, and gait training, whether it be in early childhood development or in the post-operative state. Our survey demonstrated that just over 50% of patients have tried aquatherapy (180/337). On the contrary, nearly 50% of patients have not had access to aquatherapy (43%).

### 3.5. Patient and Family Comments

**Figure 3:**
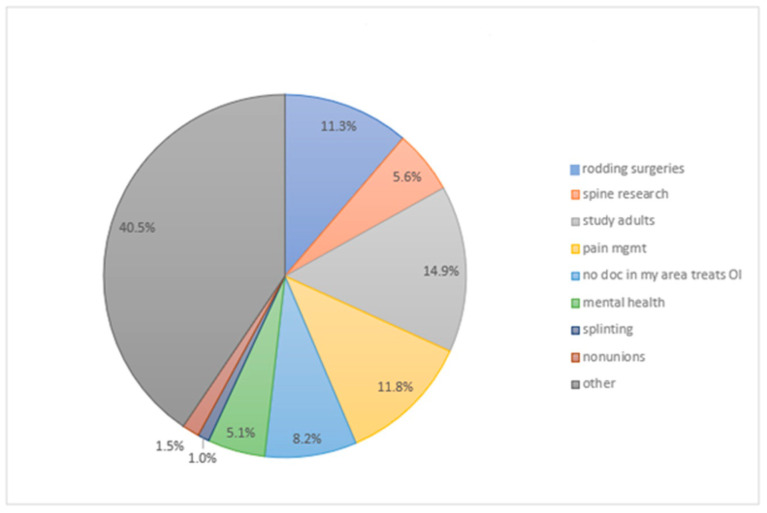
Summary of Free Response Answers.

The survey’s final question was an open-ended question requesting candid and honest comments from patients and families regarding the topic of the survey. There were 195 individual responses in this section that were grouped into eight themes (Figure 3). The most frequent comments were to focus future research on OI adults (14.9%), focus on pain management (11.8%), and focus on improving rodding surgery techniques (11.3%). Approximately 40% of the responses were more challenging to categorize into themes, and they were put in the “other” category, but there were several comments requesting additional focus on joint replacement in OI adults, joint stiffness in OI adults, and the use of ambulatory aids in OI patients.

## 4. Discussion

This survey was formed to assess orthopedic information provided to OI patients and families and to assess orthopedic concerns in the OI population. The goal of orthopedic surgeons is to have patients and families help guide us in our patient care, patient education, and research endeavors. The authors are each a part of the multidisciplinary care of children with OI at their respective institutions. Each center sees children from multiple regions of the United States and has found that depending on the location in which patients live, orthopedic care options or recommendations differ significantly. The purpose of this survey was to learn from OI patients and families both nationally and internationally with varied access to healthcare systems regarding their orthopedic experience and perceptions of access to care and the recommendations they have been given by orthopedic providers.

There are an estimated 25,000–50,000 people in the United States living with OI. The OIF has 800–900 active patients and families they serve on a routine basis. The OIF and OIFE sent the survey to their constituents. This provided both a national and international patient perspective on OI orthopedic care. The survey was also posted on social media platforms. We were pleased with the large number of respondents; 341 separate surveys were completed. A similar survey was completed and published recently in Europe for patients with rare bone diseases, 53.4% of the respondents had OI. They found that these patients and families were most interested in improving treatment and medical services and less interested in anxiety and socializing in context with their disease [20].

Many of the survey questions we asked were related to childhood orthopedic surgical interventions. However, 75% of the respondents that answered the age question (254/335) were adults. There is likely recall bias for these respondents. There have been several iterations of telescoping rods over the years. The most commonly used telescoping rod currently in North America is the Fassier–Duval rod, which started being used with increasing frequency around the year 2000 [21].

Regarding surgical intervention for long bones, only 16% of respondents recall that they have been told that they could not have surgery because they were too young. Of the 16%, 37.8% were told that <5 years was too young, 13.4% <4 years was too young, and 48.8% <3 years of age was too young for surgical intervention for fractures or deformities. Nearly 22% of respondents were told their bones were too small to have an intramedullary fixation. Unfortunately, we cannot assess from the data if this is what patients were told before or after more modern implants were available for treatment. We also are unable to assess if patients were told they were not candidates for intramedullary telescoping rods before or after bisphosphonate infusions.

The information collected helps reveal trends of the typical orthopedic advice patients and families with OI are receiving. Having the details of the survey and the patient/family’s responses will help guide future orthopedic patient-reported outcomes in the arena of OI clinical and surgical care. The goal is to have patients’ concerns and insights drive the research questions we ask in the orthopedic care of patients with OI.

We also learned that nearly 50% of patients that answered this survey have never experienced aquatherapy as part of their OI management. Unfortunately, we do not know the barriers to this type of treatment—whether it be pool availability in the area, insurance authorizations, or possibly even long wait lists.

This study provides patient and family perspectives on OI orthopedic care in a standardized way. As pediatric orthopedic surgeons who care for children with OI, we all have seen children for second opinions regarding surgical care and felt that this was an important topic to survey the OI community. We wanted to assess if other patients also experience similar advice from other surgeons regarding surgical care, post-operative care, and overall orthopedic management. We found that the families that seek second opinions from multi-disciplinary clinics are similar to the patients and families who responded to our survey.

The limitations of this survey include the self-selected population, a heterogeneous population (36% mild OI, 50% moderate OI, and 14.75% severe OI) of respondents, and survey fatigue which can occur in patients who live in the rare disease world. One other confounding variable is that most of our respondents were adults, answering questions related to when they were children. This likely has a high chance of recall bias as we were asking questions about when patients were small children <5 years of age.

## 5. Conclusions

Future directions are to utilize patient and family concerns to fuel future OI research. Critically evaluating surgical outcomes of rodding surgery based on the initial age of the surgery will be important, including investigation of secondary revision surgeries, fracture burden, and physical developmental milestones [21,22]. Another important function to be able to assess is the timing of surgery and the ultimate ability to ambulate. As developmental milestones and ambulation are multifactorial in all children, but especially in children with moderate to severe OI this is a complex topic to investigate. Montpetit et al. found that the biggest predictor of ambulation at skeletal maturity was the ability to ambulate by age 5 [22]. Another study found that initial FD femoral rodding improved OI patients’ ability to ambulate and showed significant improvements in gross motor function more than what would have been expected for normal growth and development in their patient cohort [21]. Future studies should focus on the timing of surgical intervention for deformity correction based on age and function and how this may or may not affect the ultimate ability to weight bear for transfers or ambulate at skeletal maturity.

Another future study could also evaluate aquatherapy, its use, efficacy, and how to increase access for patients across the country. Another critical factor to patients is orthopedic care as patients age out of pediatric facilities and become adults. This has been a common theme brought up by patients in this survey and recent OIF events. It is clear from the study that additional resources are needed and essential to adult patients with OI to determine best practices and care for orthopedic issues that arise during adulthood. One frequent concern that the participants of this study raised is joint stiffness, which could be researched further by adult orthopedic providers or pediatric orthopedic OI providers who can take care of adults in their pediatric centers. Currently, there are only case reports of joint replacements in adult OI patients.

## Data Availability

The data presented in this study are available on request from the corresponding author. The data are not publicly available due to patient confidentiality concerns.
